# The Kidney-Associated Microbiome of Wild-Caught *Artibeus* spp. in Grenada, West Indies

**DOI:** 10.3390/ani11061571

**Published:** 2021-05-27

**Authors:** Maria E. Ramos-Nino, Daniel M. Fitzpatrick, Korin M. Eckstrom, Scott Tighe, Julie A. Dragon, Sonia Cheetham

**Affiliations:** 1 Department of Microbiology, Immunology, and Pharmacology, School of Medicine, St. George’s University, West Indies, Grenada; 2 Department of Pathobiology, School of Veterinary Medicine, St. George’s University, West Indies, Grenada; dfitzpat@sgu.edu (D.M.F.); scheetha@sgu.edu (S.C.); 3 Larner School of Medicine, University of Vermont Massively Parallel Sequencing Facility, Burlington, VT 05401, USA; korin.eckstrom@med.uvm.edu (K.M.E.); scott.tighe@uvm.edu (S.T.); julie.dragon@med.uvm.edu (J.A.D.)

**Keywords:** *Artibeus*, bats, betaretrovirus, kidney, metagenomics

## Abstract

**Simple Summary:**

Bats are increasingly being recognized as important integrants of zoonotic disease cycles. Studying bat microbiomes could potentially contribute to the epidemiology of emerging infectious diseases in humans. Furthermore, studying the bat’s microbiome gives us the opportunity to look at the microbiome evolution in mammals. Bat microbiome studies have focused mainly on the gut microbiome, but little is known of the microbiome of the kidney, another potential source of disease transmission. Furthermore, many studies on microbiome found in the literature are based on captive animals, which usually alters the natural microbiome. Here, we analyzed kidney samples of wild-caught *Artibeus* spp., a fructivorous bat species from Grenada, West Indies, using metagenomics.

**Abstract:**

Bats are capable of asymptomatically carrying a diverse number of microorganisms, including human pathogens, due to their unique immune system. Because of the close contact between bats and humans, there is a possibility for interspecies transmission and consequential disease outbreaks. Herein, high-throughput sequencing was used to determine the kidney-associated microbiome of a bat species abundant in Grenada, West Indies, *Artibeus* spp. Results indicate that the kidney of these bats can carry potential human pathogens. An endogenous retrovirus, *Desmodus rotundus* endogenous retrovirus isolate 824, phylogenetically related to betaretroviruses from rodents and New World primates, was also identified.

## 1. Introduction

The microbiome is a key element of life. It is associated with the immune system and defense against pathogens [1,2,3], energy processing [4,5], behavior [6], mating [7,8,9], and evolution [10], among other aspects. Many studies on microbiome found in the literature are based on captive animals, which usually alters the natural microbiome. Studies on wild animals are needed to establish key players in the microbiome and determine how it affects the potential host for disease transmission and its capacity as a reservoir [10]. 

Bats (order Chiroptera, suborders Megachiroptera and Microchiroptera), encompassing 17 families and >1200 species, are the second-largest mammalian group in the world [11,12,13]. Bats’ lifestyle, including food choices, population structure, movement patterns, life span, and roosting behaviors, make them accessible to many pathogens [12,14]. However, what makes them a potential reservoir of human pathogens and other animals is the specialized immune system that allows them to carry pathogens without being affected themselves. The evolution of bats may have selected a unique set of antimicrobial immune responses that control microbial propagation while limiting self-damaging inflammatory responses [15,16,17]. Among the microorganisms detected in healthy bats are filoviruses, paramyxoviruses, and coronaviruses that cause severe diseases, such as Ebola virus disease, Marburg hemorrhagic fever, and severe acute respiratory syndrome (SARS), in humans [13,18,19,20,21,22]. The increasing rate of bat-associated infections is supported by an increasing overlap between bat and human habitats [21]. The Database of Bat-associated uses (DBatVir) (http://www.mgc.ac.cn/DBatVir (accessed on 1 May 2020)) provides updates on the virome diversity of bats, as well as ecological and epidemiological data to track bat-related transmissible diseases.

Bats play an important role in many ecosystems [23,24,25], but little is known about their microbiome and how it impacts the health and behavior of the bats in different regions. Bat-associated disease outbreaks in humans (e.g., Nipah, Hendra, SARS-Cov1, SARS-Cov2, and Ebola) have, however, stimulated research on microbiome dynamics in bats [12,26,27]. Some examples of metagenomes of bats have appeared in the literature [13,27,28,29,30,31], but more research is needed at the local level and with a specific focus on particular mechanisms to determine their potential involvement in pathogen transmission. 

Published studies on the microbiota of bats have focused on capture and euthanasia or capture and release of bats. They have used urine and fecal samples directly collected in hand from the animal [32,33,34,35,36]. Other studies have focused on the use of gut tissue or feces for the analysis of the microbiome in bats, and few studies have used urine samples for this analysis as well; however, to our knowledge, no study has focused on kidney’s microbiota for this analysis. In this study, we analyzed kidney samples of the *Artibeus* spp. population, a fructivorous bat species, from Grenada, West Indies. 

## 2. Materials and Methods

Neotropical bats were trapped on the island of Grenada, West Indies, from 2015 to 2017 using mist nets, hand nets, and a harp trap. Live capture, along with proper monitoring of traps and nets, ensured animal safety as recommended by the Animal Care and Use Committee of the American Society of Mammalogists [37]. Bats (n = 173) of the most abundant species were collected from coastal areas around the island where the majority of the human population is located. Due to the changing taxonomic status of bats in the *Artibeus jamaicensis* complex of bats and the difficulty of classifying the *Artibeus* genus by morphology alone, all potential *Artibeus jamaicensis*, *Artibeus planirostris*, and *Artibeus schwartzi* bats in this study were collectively identified as *Artibeus* spp. Bats were identified morphologically [38] and confirmed by cytochrome B PCR [39] as *Artibeus* spp.

### 2.1. Bat Processing

Live bats were transported to the necropsy laboratory at St. George’s University, School of Veterinary Medicine (SGU SVM), Grenada, West Indies, in individual opaque cloth bags to prevent post-capture cross-contamination. Bats were euthanized in the necropsy lab using isoflurane followed by thoracotomy and cardiac exsanguination while under anesthesia. Tissue samples were stored in RNAlater at −20 °C and formalin.

### 2.2. Histopathology 

Bat kidney tissues were fixed by immersion in 10% neutral buffered formalin, embedded in paraffin, sectioned at 4 μm, stained with hematoxylin and eosin (HE) and Warthin–Starry (WS) silver stain (kidneys only) using standard histological techniques, and examined by light microscopy (Nikon LV100, Microscopecentral, Feasterville, PA, USA) by a board-certified veterinary pathologist as previously described [40].

### 2.3. Total RNA Extraction and RNA-Seq

RNA was extracted from 30 mg of kidney tissue from two randomly selected bats after tissue disruption in a bead-beater (Mini Beadbeater Biospec Products, Bartlesville, OK, USA) using TRIzol (Life Technologies Cat#15596-018). Invitrogen™ Phasemaker™ Tubes (ThermoFisher Scientific Cat#A33248) were used for phase separation. RNA was DNase-treated using TURBO DNA-*free*™ (Life Technologies Cat#AM1907, Durham, NC, USA), and RNA quality was evaluated using a Bioanalyzer (Agilent 2100, Agilent Technologies. Inc, Santa Clara, CA, USA) as previously described [41].

**Figure 1:**
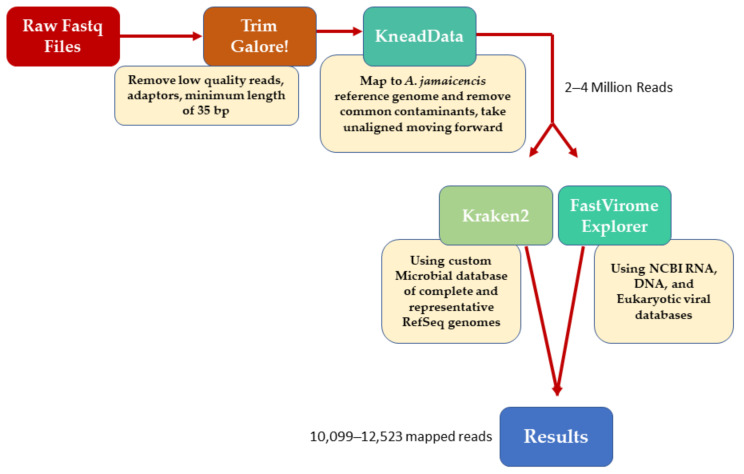
Metagenomic data analysis flow chart.

Libraries for shotgun metagenomic and meta-transcriptomic sequencing were pooled and run on a single lane of an Illumina HiSeq 2500 (Illumina, San Diego, CA, USA) Quality of raw reads was assessed using FastQC version 0.11.8; the reads were then trimmed using Trim Galore (v 0.6.4) (https://www.bioinformatics.babraham.ac.uk/projects/trim_galore/ (accessed on 1 May 2020)) to remove Illumina universal adaptors, bases with a quality score <20, and reads shorter than 35 bp. Trimmed reads were mapped to the *A. jamaicencis* reference genome (https://www.ncbi.nlm.nih.gov/genome/12026?genome_assembly_id=437954 (accessed on 1 May 2020)) using KneadData (v0.7.4) (https://huttenhower.sph.harvard.edu/kneaddata/). Reads mapping to the host was removed from further analysis, leaving 2–4 million reads per sample. Taxonomic classification was performed using Kraken2 (https://genomebiology.biomedcentral.com/articles/10.1186/s13059-019-1891-0 (accessed on 1 May 2020)) with the microbial database compiled by the Loman Lab available online (https://lomanlab.github.io/mockcommunity/mc_databases.html (accessed on 1 May 2020)). This database includes all complete and representative genomes available in RefSeq for archaea, bacteria, fungi, protozoa, viral, and UniVec_Core sequences. In addition to this, viral sequences were profiled using FastViromeExplorer (https://peerj.com/articles/4227/ (accessed on 1 May 2020)) against the NCBI DNA, RNA, and eukaryotic viral databases available at FastViromeExplorer (https://bench.cs.vt.edu/FastViromeExplorer/ (accessed on 1 May 2020)). Estimated abundance is expressed as total read counts adjusted for the segment size of detected viruses (Figure 1).

For further confirmation of results, reads matching at the genus level were filtered from the dataset and assembled using SPAdes (v3.14.0) (accessed on 2 May 2020) [42] with the parameters -k 21, 33, 55, 77, 99, 127, and with the coverage cutoff disabled due to the low abundance of certain taxa. The resulting contigs were then identified using BLASTn (Table 1). All raw data are available under the BioProject accession PRJNA638959 (https://www.ncbi.nlm.nih.gov/sra/PRJNA638959).

## 3. Results

Histopathological and postmortem examination of the bats’ kidneys suggested that the bats used in this study were healthy and showed adequate body condition, mild to moderate parasite burden, and no lesions that suggest significant overt disease within the examined organ system as described previously by us [40]. 

The resulting contigs identified using BLASTn are presented in Table 1. The metagenomic analysis of two kidney samples is presented in Table 2. 

## 4. Discussion

The microbiota found in this study, as indicated in Table 1, such as *Escherichia coli*, have rarely been mentioned in the literature in association with bats. Nowak et al. [43] investigated the presence of *E. coli* in the liver, lung, and intestine of tissues collected from 50 fruit bats of five different species (no *Artibeus* included) in the Republic of Congo. Herein, *E. coli* was detected in 60% of the bats analyzed. Although the majority of strains were assigned to phylogenetic group B2 (46.2%), which is linked with the ExPEC pathovar, the occurrence of virulence-associated genes in these strains was unexpectedly low, suggesting a lack of contact with humans or domestic animals. Future studies will be needed to characterize the kidney-associated *E. coli* population of *Artibeus* in Grenada, West Indies.

Results here and in a previous publication by our team [40] suggest that bats in Grenada, West Indies, can act as renal carriers, and this is particularly important for the epidemiology of organisms of certain specific genera, such as *Leptospira*. Leptospires colonize the renal tubules of the carrier animals and are then shed intermittently with urine. Human infection usually results from contact with this urine or from environmental sources that have been contaminated with it [44]. 

Species of the genus *Nesterenkonia* have been isolated from different ecological niches, particularly from saline habitats, and have been reported as weak human pathogens that cause asymptomatic bacteremia [45]. Most reports are associated with gut microflora, including that of Vaziri et al. [46], which reported an increased abundance of *Nesterenkonia* in the feces of patients with end-stage renal disease. No report of the presence of this organism in bats has been published to date.

The genus *Dietzia,* usually mistaken for *Rhodococcus*, has only been established recently as a separate genus. Currently, there is available evidence on the clinical significance of *Dietzia* species in the context of their potential role as human pathogens [47]. The evidence suggests there is a medical significance in the members of the genus *Dietzia*. Most of these organisms have been isolated from environmental samples, and the characterization of *Dietzia* in bats will help shed some light on these animals as potential reservoirs.

*Acidovorax* spp. similar to the fungi *Plasmopara*, are usually considered plant pathogens, and infections in humans are rare [48,49]. The presence of these organisms is probably associated with feeding habits.

Bats usually inhabit shelters with conditions favorable for fungal proliferation, including pathogenic and opportunistic species [50]. However, little is known about the fungal diversity present in bats. Herein, we found *Aspergillus* in the kidney samples. Kidneys are the most predominantly involved part of the urinary tract in invasive aspergillosis because these organisms primarily affect the lungs; however, all bats in this study were clinically healthy. The fact that *Aspergillus* was found in all samples suggests the role of bats as a carrier. Further studies will be needed to establish if this is the case.

No significant reads of viruses were found in our kidney samples, except *Desmodus rotundus* endogenous retrovirus isolate 824, as determined by the read assembly, which has been indicated in the Methods section. *Desmodus rotundus* endogenous betaretrovirus (DrERV) is present in the vampire bat *D. rotundus* population and in other phyllostomid bats; however, it is not present in all member species of this family [51,52]. DrERV is not phylogenetically related to Old World bat betaretroviruses but rather to betaretroviruses from rodents and New World primates, suggesting recent cross-species transmission [51]. Retroviruses are abundant in bats, and it is likely that they represent at least in part genomic contaminants and should therefore not directly be linked to a zoonotic potential [53].

## 5. Conclusions

The kidneys of bats are potential reservoirs for the transmission of pathogenic organisms. The fact that these organisms are in the kidneys of clinically healthy bats indicates that these animals can tolerate their presence in what should be a sterile organ. The importance of these findings in bats in the context of disease transmission is to be determined; however, some of the agents we found in these samples can be pathogenic to humans.

Another important finding of this study was the presence of a retrovirus identified in the vampire bat; however, the implication in terms of phylogenetic evolution is still to be determined.

## Figures and Tables

**Table 1:** Resulting contigs identified using BLASTn.

Organism	Sample #1	Sample #2
Bacteria	8174	6675
Fungi	4100	3083
Viruses	249	241

**Table 2:** Kidney-associated microbiota in *Artebius* spp. from Grenada, West Indies, expressed as relative estimated abundance (REA).

Organism					Sample #1	Sample #2
	Phylum	Class	Family	Genus		
Bacteria					**65.27**	**66.09**
	Proteobacteria	Gammaproteo-bacteria	*Enterobacteriaceae*	*Escherichia*	51.22	49.01
	Spirochaetes	Spirochaetia	*Leptospiraceae*	*Leptospira*	12.82	15.24
	Actinobacteria	Actinobacteria	*Micrococcaceae*	*Nesterenkonia*	0.11	0.11
	Actinobacteria	Actinobacteria	*Dietziaceae*	*Dietzia*	0.10	0.11
	Proteobacteria	Betaproteobacteria	*Comamonadaceae*	*Acidovorax*	0.09	0.08
	Firmicutes	Clostridia	*Clostridiaceae*	*Clostridium*	0.01	0.2
Fungi					**32.74**	**30.53**
		Oomycetes	*Peronosporaceae*	*Plasmopara*	31.36	30.00
	Ascomycota	Eurotiomycetes	*Aspergillaceae*	*Aspergillus*	0.34	0.42
Viruses			*Retroviridae*	Betaretrovirus *	**1.99**	**3.38**

* Endogenous retrovirus isolate 824.

## Data Availability

All raw data are available under the BioProject accession PRJNA638959 (https://www.ncbi.nlm.nih.gov/sra/PRJNA638959).
